# The Survival Effect in Memory: Does It Hold into Old Age and Non-Ancestral Scenarios?

**DOI:** 10.1371/journal.pone.0095792

**Published:** 2014-05-02

**Authors:** Lixia Yang, Karen P. L. Lau, Linda Truong

**Affiliations:** Department of Psychology, Ryerson University, Toronto, Canada; University of Leicester, United Kingdom

## Abstract

The survival effect in memory refers to the memory enhancement for materials encoded in reference to a survival scenario compared to those encoded in reference to a control scenario or with other encoding strategies [1]. The current study examined whether this effect is well maintained in old age by testing young (ages 18–29) and older adults (ages 65–87) on the survival effect in memory for words encoded in ancestral and/or non-ancestral modern survival scenarios relative to a non-survival control scenario. A pilot study was conducted to select the best matched comparison scenarios based on potential confounding variables, such as valence and arousal. Experiment 1 assessed the survival effect with a well-matched negative control scenario in both young and older adults. The results showed an age-equivalent survival effect across an ancestral and a non-ancestral modern survival scenario. Experiment 2 replicated the survival effect in both age groups with a positive control scenario. Taken together, the data suggest a robust survival effect that is well preserved in old age across ancestral and non-ancestral survival scenarios.

## Introduction

The survival effect in memory refers to a memory advantage for information processed in a survival-related context. It has been consistently demonstrated with young adults in an emerging body of studies [1]–[11]. In these studies, participants encoded unrelated words by rating how each word was relevant to a survival scenario (e.g., stranded in foreign grasslands) versus a control scenario (e.g., moving to a foreign land) or using other effective encoding strategies (e.g., rating for self-relevance or pleasantness). A subsequent memory test showed that words encoded in the survival scenario were better remembered than those encoded in other conditions (e.g., [8]).

### Survival Effect and Aging

Although the survival memory advantage has been robustly demonstrated with young adults, relatively little research has been done with older adults. Given the well-documented age-related deficits in episodic memory [12], it will be meaningful to examine whether survival encoding remains to be an effective mnemonic for older adults. Promisingly, research has suggested that older adults are able to strategically reallocate resources to focus on meaningful information, such as conceptual sources [13], gist information [14], or positive information (e.g., [15]). This processing focus shift allows older adults to perform as well as young adults on memory tasks. Similarly, it has been shown that older adults benefit to the same extent as young adults from self-referential memory processing (e.g., [16], [17]). Together, such results suggest that older adults' memory deficits could be reduced when the to-be-remembered information is important and relevant to them or when the encoding process remains meaningful into old age. This raises an interesting question: is survival-related processing still considered meaningful in older adults and thus continues to be effective in boosting their memory? If survival-related processing is deemed relevant and adaptive to all human beings [18], it would be expected that individuals of all ages, as far as memory is functioning, may prioritize information processed in a survival context, thus benefiting from this adaptive mnemonic.

However, this prediction has received mixed evidence in the literature. In support of this prediction, some recent work demonstrated the survival effect in memory with both children [19], [20] and older adults [21], [22]. For example, Aslan and Bäuml [19] used an auditory version of the survival effect paradigm and found that children between ages 4 to 10 also remembered more words rated for usefulness in a survival condition than those studied in other conditions. In addition, Nouchi [21] found that both older (*M*age  =  70.32, *SD*  =  3.31) and young adults (*M*age  =  20.19, *SD*  =  1.06) remembered more words rated in a survival scenario than those rated for self-reference. Pandeirada et al. [22] extended the survival effect to both healthy and cognitively impaired older adults. The findings suggest that survival processing enhances memory equivalently across a wide range of ages. In conflict with this prediction, more recent work found that survival processing did not benefit healthy older adults at all [23]. In this study, the researchers speculated that the lack of a survival effect in older adults was due to the fact that they have passed their prime reproductive time; thus, their roles in the social setting have adaptively shifted away from fitness-related evolutionary goals to emotionally satisfying and socially useful goals. However, this does not well explain the results of other studies that did find the survival effect in older adults [21], [22]. The discrepancy of previous findings on survival effects in later life leaves an intriguing question to be further explored. Along this line, we proposed the possibility that the control scenario (i.e., moving to a new city) used in Stillman et al. [23] was not well-matched with the survival scenario (i.e., stranded in the grasslands) on important variables, such as valence and arousal, which may have masked any survival effects in older adults. For example, it is possible that older adults' anti-negativity bias (i.e., avoidance of negative information; [15]) may have caused them to avoid the deep processing of information within a negative survival scenario relative to a less negative control scenario. This anti-negativity tendency in older adults may have counteracted and thus eliminated the survival effect in Stillman et al. [23]. Given the above, the present study aims to further examine the survival effect in young and older adults by comparing survival scenarios with well-matched control scenarios (e.g., scenarios rated as equally negative and/or arousing). A pilot study was conducted to ensure the comparability of the scenarios on factors that may affect memory such as valence, arousal, novelty, and familiarity.

### Survival Effects in Ancestral and Non-Ancestral Scenarios

It has been suggested that survival scenarios featuring our ancestors' living environment (e.g., grasslands) produce a larger survival effect than other survival scenarios featuring modern life contexts (e.g., city; [6], [11]. This effect has been interpreted through an evolutionary perspective which suggests that our memory systems are adaptively tuned to “ancestral priorities” or in other words, based on problems that our ancestors had to solve in their environments. However, this ancestral priority account has been challenged by some recent findings of equivalent survival effects for both ancestral survival scenarios (e.g., grasslands, lost in a jungle) and modern survival scenarios (e.g. zombies in a city, lost in outer space; [3], [10]), and context-unspecified survival scenarios ([24]; see [25] for a review). The discrepancies between previous findings may be due to differences in potentially important variables (e.g., valence and arousal) in the specific survival vs. non-survival scenarios used in these studies. Without a well-matched control, the question of generalizability from ancestral to modern survival contexts remains unclear. To address this issue, the current study selected two best-matched scenarios (based on pilot data) to investigate whether the survival advantage generalizes to both ancestral (i.e., stranded in grasslands) and modern (i.e., stranded on a mountain) survival scenarios in both young and older adults.

It should also be noted that in most previous studies, the traditional survival scenarios involved two components that threatened survival: lack of basic survival materials (e.g., water and food) and the presence of threat such as “predators” or “attackers”, which presumably activates the motivation for self-preservation and threat avoidance respectively [10]. Nairne and Pandeirada [6] found a significant survival effect in ancestral scenarios that did not include the threat of predators/attackers. The current study followed the same approach to focus on basic self-preservation survival by removing “predators/attackers” from the corresponding survival scenarios. This allowed us to assess the adaptive function of memory at the most primary survival level of self-preservation.

Taken together, the current study aimed to examine the following intriguing, yet understudied research questions: (1) Is the survival effect in memory, at the basic self-preservation level, well preserved in older adults as compared with young adults? (2) Does the survival memory advantage based on self-preservation motivation generalize across ancestral and non-ancestral modern survival scenarios? (3) Do all these effects persist after controlling for potential confounding variables involved in survival effects?

A pilot study was first conducted to select best-matched scenarios for the two subsequent experiments by rating and controlling for potential confounding variables (i.e., valence, arousal, novelty, and familiarity). In Experiment 1, we examined age differences in the survival effect with a within-subjects manipulation of survival (i.e., an ancestral and a modern context) and a well-matched negative non-survival (i.e., “cruise”) control scenario. In Experiment 2, we aimed to replicate the age effects we found in Experiment 1 with a well-matched positive non-survival control scenario (i.e., “lottery”). Such replication would support the hypothesis that the survival effect is a robust and overarching memory mnemonic. It would also suggest that this advantage goes beyond the age-related shift towards positive information (i.e., a processing bias favoring positive over negative information; [26]–[28]).

### The Pilot Study: Controlling for Potential Confounding Variables

Although previous work has considered some factors that potentially contribute to survival effects, such as arousal, novelty, media exposure, familiarity [2], [6], and schematic processing [11], little has been done to proactively match the control and survival scenarios on these variables. In the current work, we took the initiative to select best-matched control scenarios with a pilot rating task.

## Methods

Twenty young adults (ages 18**–**28, *M*  =  22.10, *SD*  =  2.53) and 20 older adults (ages 60 to 81, *M*  =  70.05, *SD*  =  7.53) rated eight scenarios using the Scenario Rating Scale developed in our lab (see Appendix S1), with “grasslands” (4^th^) as the ancestral survival scenario; “mountain” (2^nd^), “desert” (6^th^), and “city” (8^th^) as non-ancestral modern survival scenarios; “moving” (3^rd^) and “cruise” (5^th^) as non-survival negative control scenarios; and “vacation” (1^st^) and “lottery” (7^th^) as non-survival positive control scenarios. The scenarios were presented in two different pseudo-randomized orders, counterbalanced across participants. The ratings were based on a 9-point Likert-type scale on the following dimensions: valence, arousal, novelty and familiarity; higher ratings indicated higher levels of positive valence, arousal, novelty, and familiarity. The “grasslands”, “moving”, “vacation” and “city” scenarios were modified from previous studies [1], [11], and the other scenarios were created following the same schematic structure.

### Ethics Statement

The present study was approved by the Research Ethics Board of Ryerson University in Canada. Written informed consent was obtained from each participant.

## Results

We conducted a series of repeated-measures ANOVAs on the ratings for each dimension (i.e., valence, arousal, novelty, and familiarity) across comparison scenarios, with a specific focus on the contrasts to compare each candidate control scenario with the corresponding reference survival scenario. The purpose of the analyses was to select a best-matched control scenario for the corresponding reference scenario on each of these dimensions.

We started with three sets of overall ANOVAs involving both age (young vs. old) and scenario as factors on ratings for each of the dimensions (valence, arousal, novelty, and familiarity). The first set aimed to select a best-matched non-ancestral survival scenario. Four survival scenarios were compared: “Grasslands”, which has been traditionally used as the *ancestral* survival scenario, was compared to the other three scenarios that simulated modern *non-ancestral* environments (i.e., “mountain”, “desert” and “city”). The second set of analyses aimed to select a better-matched *non-survival* negative control scenario (“cruise” or “moving”) for the reference *modern* survival scenario (i.e., “mountain”) to be used in Experiment 1. The third set of analyses aimed to select a better-matched *positive non-survival* scenario (“vacation” or “lottery”) for the reference *modern* survival “mountain” scenario in Experiment 2.

The analyses on the most critical variables of valence and arousal did not reveal any age effects or age by scenario interactions (*p*s > .13). However, older adults consistently rated the scenarios higher in novelty than did young adults in all three sets of analyses (*p*s < .03), qualified by a significant or marginally significant age by scenario interaction in the first two sets of analyses (*p*s < .03). This was mainly driven by older adults' higher novelty ratings to the “mountain” (*M*  =  8.25) relative to the other scenarios (*M*  =  7.45, 8.10, 7.15, 7.30, and 7.25 for grasslands, desert, city, cruise, and moving scenario respectively). In contrast, young adults' novelty ratings were relatively similar across scenarios (*M*  =  7.35, 6.85, 6.70, 6.45, 6.85, and 5.10 for grasslands, mountain, desert, city, cruise, and moving scenario respectively). In addition, there was an age by scenario interaction in the third set of analyses on familiarity ratings. Although both age groups rated mountain as differentially less familiar, older adults tended to show larger differences in familiarity ratings between the “mountain” (older: *M*  =  1.95; young: *M*  =  2.85) and the “vacation” (older: *M*  =  5.70; young: *M*  =  4.80) scenarios.

Given the complexity and inconsistency of the pilot data across the two age groups and our main focus on controlling for valence and arousal – which did not show age effects or age by scenario interactions – we collapsed across age groups and reported the planned simple contrast results based on the whole sample in all three sets of analyses. Table 1 shows the descriptive statistics of the rating data and the results of the planned contrasts.

**Table 1:** Pilot study ratings.

Scenario	Valence ratings	Arousal ratings	Novelty ratings	Familiarity ratings
Grasslands^a^	2.55 (1.28)	7.33 (1.23)	7.40 (1.61)	2.25 (1.33)
Mountain^b^	2.30 (1.11)	7.10 (2.01)	7.55 (1.75)	2.40 (1.41)
Desert^b^	2.03 (1.12)**	7.45 (2.09)	7.40 (1.77)	2.78 (2.04)^†^
City^b^	3.03 (1.79)^†^	7.35 (1.42)	6.80 (1.47)*	3.20 (1.91)**
Cruise^c^	2.23 (1.29)	7.50 (1.48)	7.08 (1.73)	3.43 (2.00)**
Moving^c^	4.75 (2.12)**	6.78 (1.23)	6.18 (2.26)**	4.53 (2.48)**
Lottery^d^	8.30 (0.72)**	7.38 (1.88)	7.73 (1.54)	3.85 (2.48)**
Vacation^d^	7.15 (1.66)**	6.08 (1.67)*	5.53 (2.21)**	5.25 (1.90)**

*Note.* Each cell provides the mean score, with the standard deviation (SD) in the parenthesis. “Grasslands” was the reference scenario for selecting a best-matched modern survival scenario. ”Mountain” was the reference scenario for selecting a best-matched negative and positive non-survival scenario. ^†^ *p* < .10, *p < .05, **p <.01, with all significant effects dictating the simple planned contrast between each scenario with its corresponding reference scenario. For example, the effects shown in the “valence ratings” column suggest that “city” marginally differs from “grasslands” (*p* < .10), whereas “moving”, “vacation”, and “lottery” differ from “mountain” (*p*s < .001) in valence ratings. ^a^ancestral survival scenario, ^b^modern survival scenario, ^c^negative non-survival scenario, and ^d^positive non-survival scenario.

In the first set of simple contrast analyses, all of the included survival scenarios (grassland, mountain, city, and desert) were rated as negative in valence (valence ratings ≤ 3.03). The specified simple planned contrasts using “grasslands” as a reference condition showed that “city” was rated marginally less negative in valence (*p*  = .06), significantly more familiar and less novel (*ps* < .04) than “grasslands”. “Desert” was rated more negative in valence than “grasslands” (*p* < .01). However, “mountain” did not differ from “grasslands” on any of the dimensions (*p*s > .13) and thus it was chosen as the best-matched *modern* survival scenario.

In the second set of simple contrast analyses, all the included scenarios (mountain, cruise, and moving) were rated as negative in valence (ratings ≤ 4.75), but the “cruise” and “moving” scenarios did not include a survival component (e.g., lack of basic survival materials). The simple planned contrasts showed that “moving” was significantly less negative, less novel, and more familiar than “mountain” (*p*s < .01). “Cruise” was more familiar than “mountain” (*p*  = .003) but did not differ from “mountain” on any of the other dimensions (*p*s > .14); therefore “cruise” was chosen as a better-matched *non-survival* control scenario.

In the third set of simple contrast analyses, both “vacation” and “lottery” were rated positive in valence (ratings ≥ 7.15), and they were both rated more positive and more familiar than “mountain” (*p*s < .001). Nevertheless, “lottery” was chosen as the better-matched control because it did not differ from “mountain” on arousal and novelty (*p*s > .53) whereas “vacation” was rated lower in arousal and novelty (*ps* < .05).

## Experiment 1

In Experiment 1, we adopted a within-subjects manipulation of survival scenarios (“grasslands” and “mountain”) and a well-matched negative control scenario (“cruise”) to examine survival memory effects in young and older adults.

### Participants

Thirty-six older adults (ages 65**–**87, *M*  =  73.61, *SD*  =  6.24) in the local community and 36 young adults (ages 18**–**29, *M*  =  22.14, *SD*  =  3.03) in an undergraduate psychology participant pool were recruited to participate in the study. Exclusion criteria included a prior history of neurological or major psychiatric conditions, uncontrolled diabetes or cardiovascular conditions, or a low vocabulary (i.e., scored below 20 on the Shipley Vocabulary test; [29]). Young participants started to learn English before the age of 6. Older participants started to learn English at least 40 years prior to the test date. Three older adults were replaced due to technical problems. All older adults scored above 26 (*M*  =  28.86, *SD*  =  1.15) on the Mini Mental State Exam (MMSE; [30]), a screening measure for potential dementia-related cognitive impairments. The sample characteristics are displayed in Table 2.

**Table 2:** Participant characteristics.

Measures	Experiment 1		Experiment 2	
	Young	Older	Young	Older
Gender ratio^a^	9∶27	8∶28	7∶17	1∶23
Age	22.14 (3.03)	73.61 (6.24)	19.13 (2.07)	73.08 (5.49)
Years of education	14.94 (1.92)	16.53 (3.28)	12.58 (1.06)	16.58 (2.89)
Digit Symbol	90.44 (14.17)	60.89(10.70)	85.21 (10.27)	65.25 (12.20)
Shipley vocabulary	27.36 (3.29)	37.28 (2.35)	26.29 (3.90)	37.25 (2.27)
BAI	14.64 (10.61)	5.94 (6.29)	18.08 (10.08)	5.50 (4.65)

*Note.* Each cell, except those for gender ratio, provides the mean score, with the standard deviation (SD) in the parenthesis. ^a^male/female gender ratio. BAI  =  Beck Anxiety Inventory.

### Ethics Statement

The present study was approved by the Research Ethics Board of Ryerson University in Canada. Written informed consent was obtained from each participant.

### Stimuli and Design

A word pool of 48 target words and nine practice words was chosen from Nairne et al. [1], Weinstein et al. [11], and the Affective Norms for English Words (ANEW; [31]). They were divided into three sets of 16 pre-randomized target words and three practice words. The three sets of target words were matched on concreteness, word frequency and word length. For the words selected from the ANEW, we attempted to select words in a neutral range (4.2**–**5.7) based on the ANEW norms (i.e., a 9-point scale ranging from 1 to 9, with higher ratings indicating more positive valence). Each set was presented equally often in each of the three distinct scenarios presented in three counterbalanced encoding blocks. This resulted in nine counterbalanced versions of presentation.

A 2 (age: young vs. older) × 3 (scenario: ancestral survival, non-ancestral survival, non-survival control) mixed design was adopted, with scenario as a within-subjects variable. During encoding, participants rated a series of words for their relevance to one of the three scenarios (i.e., *ancestral* survival “grasslands”, *non-ancestral* survival “mountain” or *non-surviva*l control “cruise”). Appendix S1 shows the specific wording of each scenario.

### Procedure

The memory encoding task consisted of rating words for their relevance to different scenarios. The task was programmed in E-prime 1.1 and involved three blocks, each requiring participants to rate 16 words for their relevance in an encoding scenario. Each encoding trial began with a fixation sign “+” presented at the center of the screen for 1 s, which was then replaced by a word that was presented for 5 s. A corresponding 5-point rating scale appeared below each word, with 1 meaning “totally irrelevant” and 5 meaning “extremely relevant”. Participants rated each of the 16 words, using this scale, for its relevance to the encoding scenario in each block. Immediately following the last encoding block, participants completed a 2-minute perceptual-motor speed task, the Digit Symbol task [32], as a nonverbal filler task. A free recall test followed in which participants were given a piece of blank paper and told to write down as many words as they could remember from the encoding task. Participants were given up to 10 minutes for the recall task.

Following this memory task, participants were asked about their awareness of the recall memory test on a 3-point scale (1  =  completely unaware, 2  =  somewhat aware, and 3  =  aware). Two participants reported being somewhat aware but did not try to remember the words, suggesting that the encoding task was largely incidental. Analyses conducted without these two participants did not alter the results. Participants also rated how often they watched survival-themed movies or television shows on a 5-point scale (1  =  never, 2  =  rarely, 3  =  sometimes, 4  =  often and 5  =  all the time). Additionally, they rated how often they have been on a cruise based on the same 5-point scale. None of these three measures correlated with the memory scores for each of the three scenarios (*r*s < .15, *p*s > .23). Participants then completed a set of paper-and-pencil questionnaires and tests, including the Shipley vocabulary test, the Beck Anxiety Inventory (BAI; [33]), and a background questionnaire. Finally, the MMSE [30] was administered to older adults. All participants were then debriefed and compensated with one course credit (for young adults) or $10/hour (for older adults).

## Results

### 

#### Free recall

**Figure 1:**
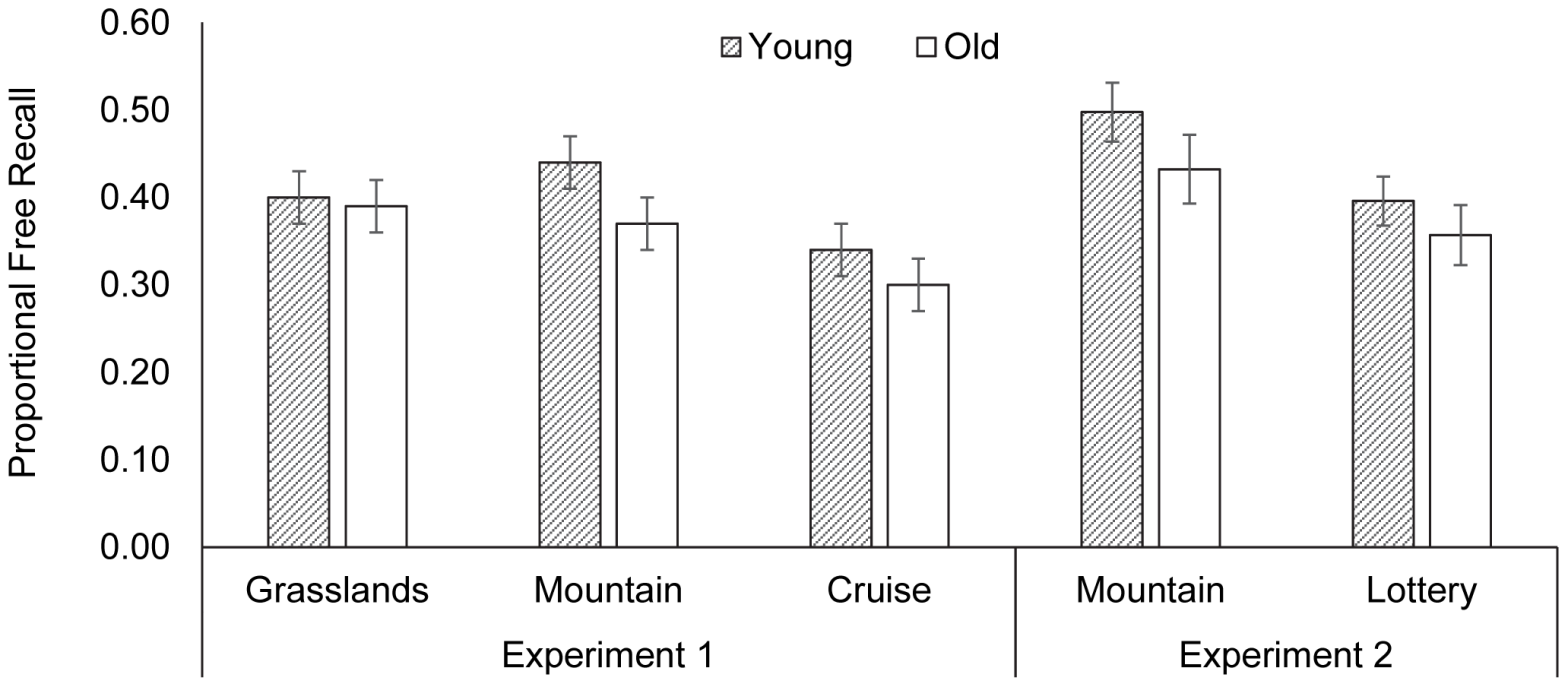
Proportional free recall across conditions and age groups in Experiments 1 and 2. Error bars denote mean standard errors.

The proportional recall of Experiment 1 is displayed in the left panel of Figure 1. A 2 (Age) × 3 (Scenario) mixed ANOVA revealed a significant scenario effect, *F* (2, 140)  =  6.52, *p*  =  .002, η^2^  =  0.09. Pairwise comparisons with Bonferroni corrections revealed higher recall for words encoded in the “grasslands” (*M*  =  0.40, *SD*  =  0.17) and “mountain” scenarios (*M*  =  0.40, *SD*  =  0.17) than in the “cruise” scenario (*M*  =  0.32, *SD*  =  0.17), *p*s < .02. “grasslands” and “mountain” did not differ, *p*  =  1.00. There were no main effects of age or interactions (*p*s > .16).

The results replicated the survival effect by showing better memory performance in both survival scenarios (“grasslands” and “mountain”) compared to the non-survival control scenario (“cruise”). These findings also suggested that older and young adults benefited equivalently from the survival effect in both ancestral and non-ancestral modern survival contexts.

#### Ratings and rating response times (RTs)

The ANOVA on the ratings revealed only a significant scenario effect, *F*(2, 140)  =  19.75, *p* < .001, η^2^  =  0.22. Pairwise comparisons with Bonferroni corrections revealed that words were rated higher in both “grasslands” (*M*  =  3.19, *SD*  =  0.61) and “mountain” scenarios (*M*  =  3.25, *SD*  =  0.66) than in the “cruise” scenario (*M*  =  2.77, *SD*  =  0.71), *ps* < .001. The ratings did not differ between the “grasslands” and the “mountain” scenarios, *p*  =  1.00. The rating RTs were trimmed by removing those beyond 2.5 standard deviations away from the mean (0.57% of the trials). The ANOVA on the rating RTs revealed a main effect of age only, *F*(1, 70)  =  22.31, *p* < .001, η^2^  =  0.24, with older adults (*M*  =  2640.84 ms, *SD*  =  385.53) being slower than young adults (*M*  =  2163.22 ms, *SD*  =  468.50).

#### Congruity effect

Considering that the ratings showed a similar scenario effect pattern as the memory scores, we conducted further analyses to examine a possible congruity effect (i.e., better memory for items that were congruent with the way they were processed, which would result in better memory for words rated more relevant to a survival theme, as compared to a non-survival control; [34], [35]).

Three analyses were conducted. The first one was a subject-based 3 (scenario) × 5 (rating: 1, 2, 3, 4, and 5) ANOVA on recall. This analysis involved 19 participants who provided all the five ratings. The results showed a significant main effect of scenario, *F*(2, 36)  =  4.30, *MSE*  =  0.15, *p* < .05; and a main effect of rating, *F*(4, 72)  =  4.57, *MSE*  =  0.09, *p* < .01. However, the interaction was not significant (*p*  =  .21), suggesting that the two effects were largely independent, with a similar survival advantage across each rating. The second analysis examined the survival effects in a subject-based 3 (scenario) × 2 (age: young vs. older) ANOVA on recall after excluding participants (3 young and 8 older) who provided substantially different ratings across the three scenarios (> 1.5 in ratings), the scenario effect was marginally significant, *F*(2, 118)  =  2.95, *MSE*  =  0.02, *p*  =  .06. Post-hoc simple contrasts showed better recall in the “grassland” than “cruise” (*p*  =  .06), and in the “mountain” than “cruise” condition (*p* < .05). This suggests that the survival effect remained even after artificially controlling for different ratings across scenarios. Furthermore, at the subject level, there were no correlations between ratings and recall (*p*s > .33) within each scenario, suggesting that participants who gave higher ratings did not necessarily have a better recall. Taken together, these results suggest that the survival effect persist beyond the congruity effect.

The third analysis was an item-based 2 (recall status: recalled vs. non-recalled) × 3 (scenario) ANOVA on the average ratings. The main focus was to examine whether the successfully recalled items were also rated higher than those failed to be recalled, and how this effect varied across survival and non-survival scenarios. The results showed a significant scenario effect, *F*(2, 136)  =  17.19, *MSE*  =  0.47, *p* < .001; and an effect of recall status, *F*(1, 68)  =  61.79, *MSE*  =  0.44, *p* < .001. However, the interaction was not significant (*p*  =  .42), suggesting that the two effects were largely independent, with higher ratings for recalled than non-recalled words in both survival and non-survival scenarios. In addition, we should point out that the word lists were counterbalanced so each word was equally likely to be rated in each scenario. This indicates that even the same words tended to be rated more relevant to a survival than a control scenario. For example, ‘mirror’ would not be considered very useful in the “cruise” scenario, but it could be viewed as very useful to start a fire in the “grassland” scenario. Therefore, the congruity effect is not item-specific and may be independent of the survival processing. Any items rated higher are more likely to be recalled, regardless of the encoding scenarios.

## Experiment 2

Experiment 2 aimed to replicate whether the age equivalent survival memory advantage evidenced in Experiment 1 with a negative non-survival control scenario could be generalized to a highly positive non-survival scenario.

### Participants

Twenty-four young adults (ages 18**–**27, *M*  =  19.13, *SD*  =  2.07) from an undergraduate psychology participant pool and 24 older adults (ages 66**–**83, *M*  =  73.08, *SD*  =  5.49) from the community were recruited. They were screened with the same exclusion criteria as in Experiment 1. All older participants scored above 26 (*M*  =  28.88, *SD*  =  1.15) on the MMSE. The sample characteristics are displayed in Table 2.

### Ethics Statement

The present study was approved by the Research Ethics Board of Ryerson University in Canada. Written informed consent was obtained from each participant.

### Stimuli and Design

Thirty-two target words and six practice words were randomly selected from two of the three lists used in Experiment 1. The words were assigned into two lists, each with 16 pre-randomized target words. Each word list was rated in either the survival “mountain” scenario or the positive non-survival “lottery” scenario on the same 5-point scale used in Experiment 1. The order of the two scenario blocks and the two word lists were counterbalanced across participants, resulting in four counterbalance conditions.

A 2 (Age) × 2 (Scenario) mixed design was adopted. The “mountain” scenario was the same as that used in Experiment 1. The exact wording for the positive non-survival control scenario (i.e., “lottery”) can be found in Appendix S1.

### Procedure

We followed the same procedure as in Experiment 1. We also administered the same post-memory questionnaires (awareness of subsequent memory test and frequency of viewing survival-themed shows). Again, these did not correlate with memory scores (*r*s < .16, *p*s > .28). In addition, we asked participants to rate how often they played the lottery (1  =  never, 2  =  rarely, 3  =  sometimes, 4  =  often and 5  =  all the time). This also did not correlate with recall in the “lottery” scenario (*r*  =  .19, *p*  =  .19)

## Results

### 

#### Free recall

The proportional recall in Experiment 2 is displayed in the right panel of Figure 1. A mixed 2 × 2 ANOVA revealed a significant scenario effect, *F*(1, 46)  =  8.56, *p*  =  .005, η^2^  =  0.16, with better recall for words encoded in the survival “mountain” scenario (*M*  =  0.46, *SD*  =  0.18) than in the “lottery” scenario (*M*  =  0.38, *SD*  =  0.15). The main effect of age and the interaction were not significant, *p*s > .17. The results thus replicated the age-equivalent survival effects found in Experiment 1 and previous work [21]–[22]. The positive valence of the non-survival scenario did not appear to reduce the survival effect in older adults.

#### Ratings and rating RTs

The ANOVA on ratings showed a main effect of scenario, *F*(1,46)  =  53.16, *p* < .001, η^2^  =  0.54, with higher ratings in the “mountain” scenario (*M*  =  3.23, *SD*  =  0.73) than in the “lottery” scenario (*M*  =  2.32, *SD*  =  0.66). The main effect of age was also significant, *F*(1, 46)  =  4.66, *p*  =  .04, η^2^  =  .09, with higher ratings for older adults (*M*  =  2.94, *SD*  =  0.55) than for young adults (*M*  =  2.61, *SD*  =  0.50).

RTs were trimmed in the same way as in Experiment 1, which resulted in 0.46% of the trials being discarded. The ANOVA on rating RTs revealed a main effect of age, *F*(1, 46)  =  21.08, *p* <.001, η^2^  =  .31. Older adults (*M*  =  2559.03 ms, *SD*  =  500.86) were slower than young adults (*M*  =  1949.65 ms, *SD*  =  414.74).

#### Congruity effect

The same three analyses as those in Experiment 1 were conducted. The subject-based analyses revealed similar patterns. One exception was that the scenario effect was absent in the first 2 (scenario) × 5 (rating) ANOVA (including 16 participants who made all the 5 ratings). The 2 (scenario) × 2 (age) ANOVA on recall, after excluding participants (2 young and 9 older) who provided substantially different ratings across scenarios (> 1.5 in rating), revealed that the scenario effect remained significant, *F*(1, 35)  =  7.31, *MSE*  =  0.02, *p*  =  .011. Similar to Experiment 1, the ratings did not correlate with recall (*p*s > .39) at the subject level. Again, these results suggest that the survival effect, if any, persists beyond the congruity effect.

Similarly, the ANOVA on average ratings showed a significant scenario effect, *F*(1, 46)  =  45.43, *MSE*  =  0.80, *p* < .001; and an effect of recall status, *F*(1, 46)  =  37.60, *MSE*  =  0.28, *p* < .001; but the interaction was not significant (*p*  =  .12), with recalled words being rated higher than non-recalled ones in both scenarios. Again, these results suggest that the congruity effect is not item-specific and tends to be independent of the survival processing context.

### General Discussion

The current study served as an initial effort to use a set of well-controlled scenarios to examine whether the survival memory advantage is maintained in old age, in comparison with a well-matched negative or positive non-survival control scenario, and whether it could be found across both ancestral and modern survival contexts.

### Survival Effect and Aging

Consistent with a previous finding [21], we found an age-equivalent survival-processing memory advantage. These results suggest that survival processing is preserved as an effective mnemonic strategy for older adults, in comparison with processing in a non-survival scenario or other deep encoding strategies (e.g., self-reference; [21]). This finding adds to the literature on age-invariant survival memory advantage (e.g., [19]–[22]). Taken together, these findings suggest a robust survival effect that emerges early in childhood and is preserved into later life. As reviewed in Howe and Otgaar [18], although developmental invariance may not critically define the adaptive nature or evolutionary origins of the survival-related mnemonic benefit, it at least supports the argument that the proximate mechanisms underlying the survival effect seems to emerge early in life and remains robust at later ages. These proximate mechanisms may include elaboration, richness and distinctiveness processing, item processing, relational processing, and self-referential processing [18], [36], [37]. We speculate that goal-directed processing pertaining to self-preservation may be relevant and adaptive for people of all ages, as long as memory is functioning. Furthermore, the prominent goal of maintaining survival is socially meaningful and emotionally arousing, and thus is consistent with older adults' prioritized social and emotional goals (e.g., [15]). In this context, both age groups may have engaged in elaboration and deep encoding to enhance memory for information in the survival conditions. Based on the Selection-Optimization-Compensation model [38], older adults may strategically reallocate their limited resources to optimize their survival-related processing by engaging in deep encoding, given its prioritized relevance. This form of deep encoding may boost item-specific and relational elaboration, thus leading to age-equivalent survival effects in memory [36].

However, the lack of age differences in the survival effect in our study and Nouchi [21] is in contrast with the findings of Stillman et al. [23], who did not find evidence of a survival effect in older adults across three experiments. We speculate that the discrepancies may be due to the specific scenarios used in their study. As described in the introduction, Stillman et al. [23] used a moving scenario as a non-survival control. Our pilot data showed that the moving scenario was viewed as less negative and more familiar relative to the survival scenario. Additionally, a between-subjects design in their Experiment 1 – in which participants received only negative scenarios – may have placed older adults at a disadvantage. Based on a hypothesized age-related anti-negativity bias [15], [39], older adults may have avoided the deep encoding of information within a purely negative survival context. This tendency to avoid negative information may have overwritten the survival effect. In a within-subjects design (Experiment 3; [23]), the results showed a tendency for slightly better memory (*M*  =  .37) in the survival than in the control (*M*  =  .34) condition; however, this difference did not reach statistical significance. Together, the use of a between-subjects design in Experiment 1 and the use of a moving scenario may have limited the ability to demonstrate the survival effect in older adults in Stillman et al. [23]. With a within-subjects design and a better matched non-survival control scenario, the current study revealed robust survival effects in both age groups, across both ancestral and non-ancestral modern survival scenarios, and when compared to both negative and positive non-survival contexts. Our data suggest that the survival effect is well-preserved in old age, at least in a within-subjects manipulation of encoding scenario context and at a basic survival level of self-preservation in the absence of external threats (e.g., from predators). Nevertheless, further studies are needed to examine the specific conditions that may or may not show age differences in survival effects.

Surprisingly, we did not find any age differences in overall recall performance. This may be because the words in our study were all rated in an imagined emotionally-arousing scenario. This may have strengthened the inter-item relationship of the word lists and thus reduced output interference [40]. Given that older adults are differentially more vulnerable to interference [41], the reduced output interference may have boosted the memory performance of older adults. Furthermore, Otgaar and Smeets [20] have also suggested that survival processing may produce gist-based processing. This type of processing is generally favored by older adults [14] and may have benefited their memory performance. The lack of survival effects in the face recognition experiments by Savine, Scullin and Roediger III [42] supports this gist-based account because face recognition involves the recollection of perceptual details. In addition, if survival processing is equally meaningful and holds significant implications for both age groups, this would also minimize age differences in memory performance. Consistent with this argument, it has been shown that older adults performed as well as young adults on memory tests for words rated for their relevance to a scenario [23], as well as memory for meaningful value-based conceptual sources (e.g., true vs. false statements) despite an age-associated decline in memory for perceptual sources (e.g., male vs. female speakers; [13]. Furthermore, it has been evidenced that older adults are able to use prior knowledge and schematic support to boost their memory. For example, although older adults were able to remember fewer unrealistic prices for grocery items than young adults, they remembered a similar number of items at realistic prices compared to young adults [14]. Survival processing might be another example in which the use of schematic processing helps support older adults' memory performance. Additionally, it is also possible that older adults' longer rating RTs may have allowed them to deeply process the stimuli and thus boosted their memory performance to be equivalent to that achieved by young adults. Consistent with this result, Stillman et al. [23] also found that older adults in their study were slower at rating than young adults (in the full attention condition, Experiment 1) and an age-equivalent recall performance. However, we note that age-related slowing is a commonly reported finding in cognitive aging research [43]; thus, it is possible that longer rating times in older adults may simply reflect their generally slower processing speeds and may not contribute qualitatively to memory encoding. Finally, we also acknowledge the possibility of insufficient statistical power to detect an age group effect. The sample sizes of the two experiments were determined based on a-priori power analyses to aim for a minimum power of .77 to detect a within-between interaction effect size of .20 at α level of .05. Using the same parameters, post-hoc power analyses showed high power (.98 in Experiment 1 and .81 in Experiment 2) to detect the within-between interaction (which is the main focus of the current study), but low power (.54 in Experiment 1 and .35 in Experiment 2) to detect an age group effect [44]. All these factors may have contributed to the age-equivalent memory performance.

Although it has been demonstrated that older adults use controlled strategies to prioritize positive information [45], our results showed age-equivalent survival effect in memory even when the non-survival control scenario was positive in valence (Experiment 2). This finding replicated earlier results of significant survival effects with a positive control (i.e., vacation scenario; [4], [8]) and extended the results to older adults. This further suggests that survival-related processing is a robust and highly prioritized form of processing that is well-preserved into old age and may even go beyond the positivity effect in older adults.

### Generalizability of Survival Effect

The results of the current study not only replicated the well-documented survival effect [1], [2], [6]–[8], [20] but also demonstrated equivalent survival effects in both an ancestral survival context (i.e., “grasslands”) and a non-ancestral modern survival context (i.e., “mountain”). This finding appears to contrast previous work [6], [11] in which a survival effect in ancestral survival scenarios is greater relative to modern survival scenarios. However, the lack of the ancestral priority effect is largely consistent with some recent work [3], [10], [24] and thus added to literature suggesting that the survival memory advantage is not domain-specific, as it could be generalized beyond the ancestral survival context (e.g., “grasslands”; see [25] for a review). Together, this suggests that survival effects are not limited to the ancestral survival scenarios (e.g., “grasslands”). Both experiments in the current study revealed significant survival effects for a modern survival scenario (i.e., “mountain”) over a non-survival control. Similarly, Soderstrom and McCabe [10] found better recall for four survival scenarios relative to a pleasantness rating control condition (i.e., the survival effect), but this effect did not differ between the modern (city) and ancestral (grasslands) scenarios. In addition, Kostic and colleagues [3] extended the survival effect to other contexts that were not related to human evolution (e.g. lost in outer space/at sea), suggesting that survival effects could occur in a wide range of survival-relevant situations and that ancestral relevance is not crucial for this effect to occur. This may suggest that the survival effect in memory could be driven by survival-related proximate mechanisms, such as deep elaborative encoding, that are adaptive and functional even in the absence of ancestral priority.

Finally, the analyses on possible congruity effects added to existing literature [1], [35] by showing that the survival effect cannot be fully explained by the congruity effect. The survival advantage for recall appears to be robust, and generalizes across items rated similarly and also for participants who provided similar ratings. The item-based analysis showed that the higher the rating of an item, the more likely to be recalled subsequently, regardless of the survival context of the encoding scenarios. This suggests that the survival effect persists beyond the congruity effect, because it is not item-specific and tends to be independent of the survival processing. Although the congruity effect was found by Butler et al. [34], it has been challenged by some other studies (e.g., [35]). Consistently, previous studies also revealed a survival effect despite the higher relevance ratings for the non-survival than the survival condition [1].

### Limitations

One limitation of this study is that the “cruise” scenario (used as a non-survival control scenario) may imply that one is not socially isolated in the situation. As such, this scenario has an additional social aspect that is not present in the survival scenarios. However, Kostic et al. [3] found that social isolation was not a crucial factor in the survival effects.

The pilot study did not show consistent scenario ratings across the two age groups. Despite careful piloting to ensure our scenarios were equivalent on valence and arousal, the non-survival scenarios (“cruise” and “lottery”) were rated as more familiar than the modern survival scenario “mountain”. One may expect better memory performance when encoding in relation to more familiar scenarios because the schemas used to elaborate on the words would be more readily available than in less familiar scenarios [11]. However, according to Nairne and Pandeirada [6], although the city scenario was rated as more familiar than the grassland scenario, memory was poorer for words encoded in this scenario relative to those encoded in the grasslands scenario. In addition, post-hoc analyses showed the differences in ratings given to ancestral versus modern scenario on the “unusual” dimension did not correlate with the differences in recall between two scenarios (*r*  =  .067). Furthermore, the survival effect continued to show in participants who rated the two scenarios equally “unusual” [6]. Similarly, we found that memory for words rated in the less familiar modern survival scenario was better recalled than those rated in the more familiar modern non-survival scenarios. In addition, even though older adults rated the mountain scenario as more novel and less familiar in the pilot study, the two age groups did not differ in survival effects on memory performance. Together with previous findings [6], our results suggest that neither novelty nor familiarity played a critical role in the survival effects.

In conclusion, together with previous work (e.g., [21], [22], [25]), the current study provided evidence that the survival effect in memory appears to persist into old age and could be generalized to non-ancestral modern scenarios.

## Supporting Information

Appendix S1 Scenario Rating Scale.
(DOCX)
