# Supplementary figures and images for: The Survival Effect in Memory: Does It Hold into Old Age and Non-Ancestral Scenarios?
Source: PLoS One. 2014 May 2;9(5):e95792. doi: 10.1371/journal.pone.0095792 (PMC4008592; doi:10.1371/journal.pone.0095792)

Appendix S1


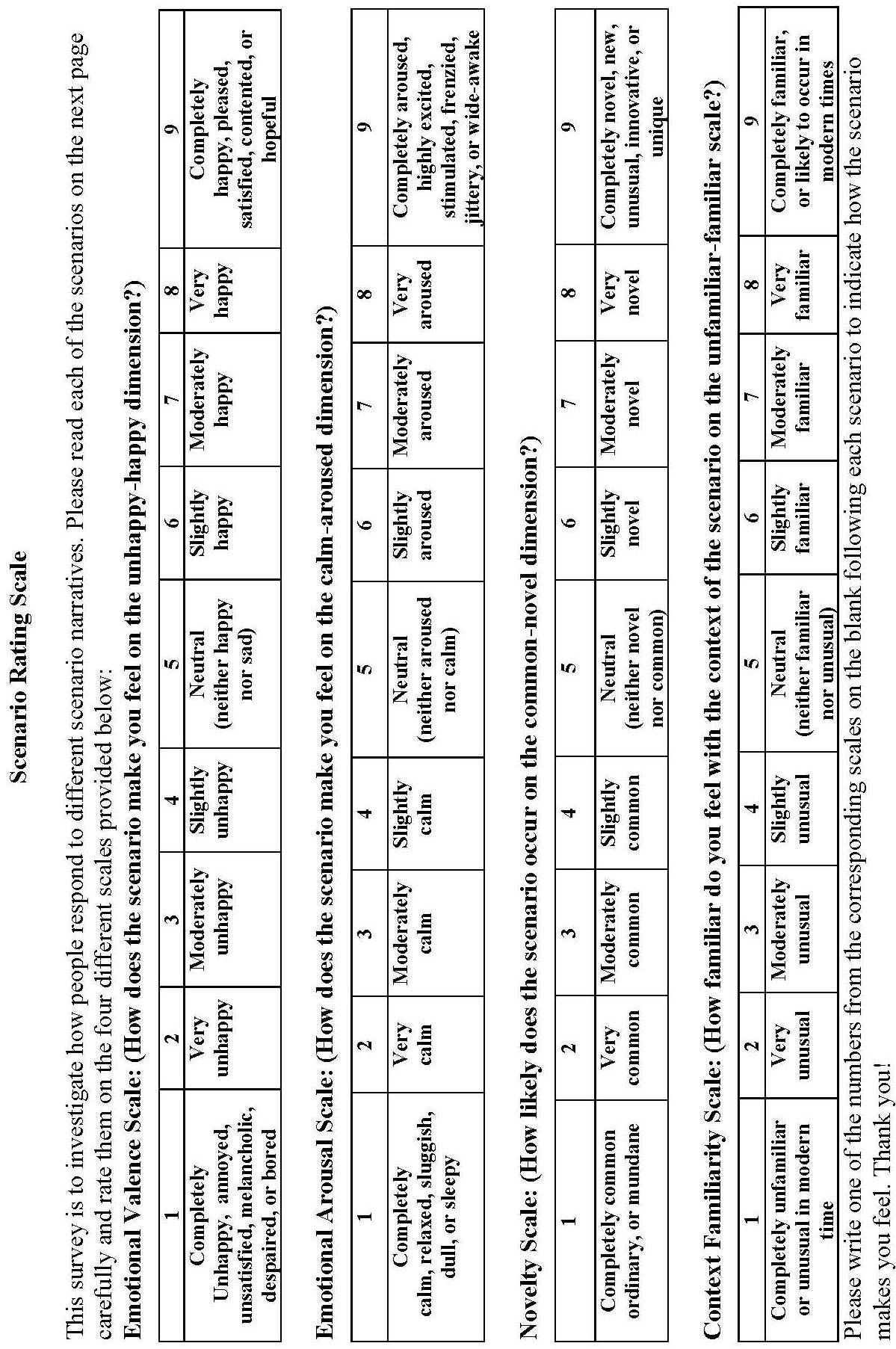


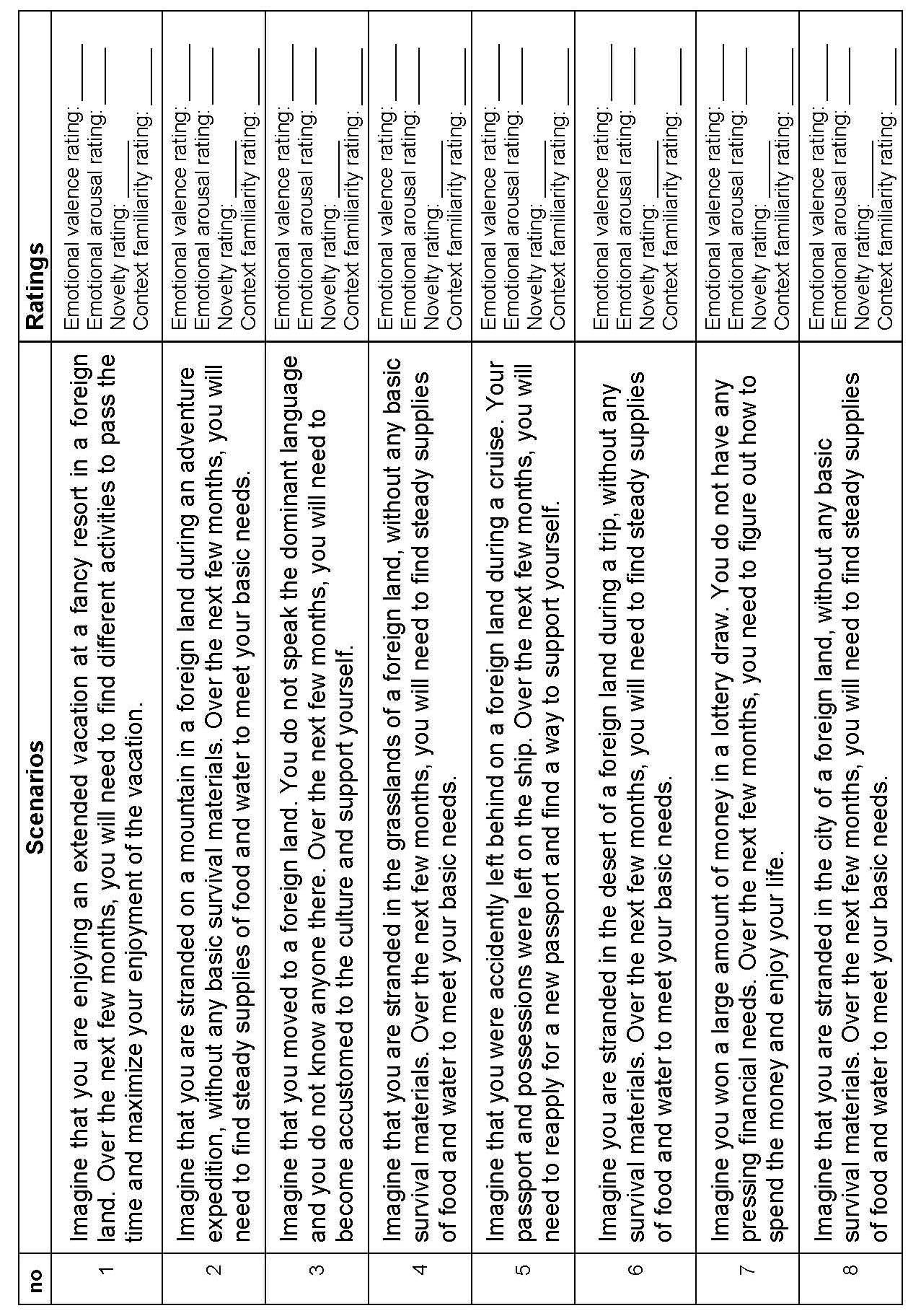

Supplement: Appendix S1 — Scenario Rating Scale. (DOCX) [file pone.0095792.s001.docx]
